# From Transnational Training to Local Capacity: The Sultanate of Oman–South Africa Split‐Funnel Model for Genetic Counseling Workforce Development

**DOI:** 10.1002/jgc4.70315

**Published:** 2026-11-03

**Authors:** Chantel van Wyk, Zandre Bruwer, Khalsa Al‐Kharusi, Nakita Laing, Tina‐Marié Wessels

**Affiliations:** ^1^ Genomics Department Sultan Qaboos Comprehensive Cancer Care and Research Center (SQCCCRC), and University Medical City (UMC) Muscat Sultanate of Oman; ^2^ Genetics and Genomics Department College of Medicine and Health Sciences, United Arab Emirates University (UAEU) Al Ain United Arab Emirates; ^3^ Genetic and Developmental Medicine Clinic Sultan Qaboos University Hospital (SQUH), and University Medical City (UMC) Muscat Sultanate of Oman; ^4^ Department of Paediatrics and Child Health Red Cross War Memorial Children's Hospital and University of Cape Town Observatory South Africa; ^5^ Division Human Genetics, Department of Pathology, Faculty of Health Sciences University of Cape Town (UCT) Cape Town South Africa

**Keywords:** clinical training, cultural adaptation, genetic counseling, transnational education, workforce development

## Abstract

As genomic technologies become increasingly integrated into healthcare worldwide, the demand for culturally and linguistically competent genetic counselors is rapidly expanding. In many regions, however, demand for these professionals far exceeds the availability of local training programs. Traditional solutions, reliance on non‐local expatriate professionals or sending trainees abroad, are often costly, logistically complex, and poorly aligned with local sociocultural contexts. In the Sultanate of Oman, where high rates of consanguinity contribute to a disproportionately elevated burden of genetic and congenital disorders, the need for a locally trained workforce is particularly urgent. This descriptive professional issue report critically reflects on a transnational training model developed through a partnership among Sultan Qaboos University Hospital (SQUH), Sultan Qaboos Comprehensive Cancer Care and Research Center (SQCCCRC) in the Sultanate of Oman, and the University of Cape Town (UCT) in South Africa to address these structural gaps. The model, conceptualized as a “Split‐Funnel Model,” integrates international academic training with sustained, supervised in‐country clinical practice. Trainees completed intensive academic blocks and clinical training at UCT while delivering clinical care in Oman, an arrangement intended to support the development of clinical expertise and culturally grounded practice. A central feature of this model is “system code‐switching,” defined as the adaptation of Western‐derived counseling frameworks to culturally responsive, collectivist, and family‐centered healthcare contexts. During the model's operation, Oman's genetic counseling workforce and service capacity expanded from a single non‐Omani‐led clinic to three national centers staffed by five qualified Omani genetic counselors; however, the descriptive design does not establish that the training model caused this growth. This experience suggests that a distributed transnational approach may be feasible and potentially valuable for developing a culturally competent genetic counseling workforce without established local academic infrastructure. Even though the report is based on retrospective programmatic observations of five trainees within one partnership, it does not establish the model's effectiveness, scalability, or transferability. However, the model may still offer practical guidance for countries seeking to build a locally embedded genetic counseling workforce before establishing an independent domestic training program.

## Abstract (highlights)

**What Is Known About This Topic:**

- Establishing culturally competent genetic counseling services in developing regions is hindered by a lack of local academic training programs, which often forces an unsustainable reliance on non‐local clinicians and medical interpreters or on sending locals abroad for training.

**What This Paper Adds to the Topic:**

- This paper describes a transnational approach that combined concentrated academic training in South Africa with sustained clinical training in the Sultanate of Oman. It identifies practical lessons concerning distributed supervision, pre‐enrollment preparation, student progression, and “system code‐switching” across linguistic, cultural, ethical, and healthcare contexts.

## Plain Language Summary

Many countries urgently need genetic counselors who speak the local language and understand the culture, but they don't yet have university programs to train them. This paper looks at a creative solution: a shared training program. Students from the Sultanate of Oman took their university classes in South Africa but completed hands‐on training with real patients back home in Oman. Completing practical clinical training in their home country enabled students to provide counseling directly in Arabic, reduce reliance on interpreters, and adapt their approaches to the local cultural, religious, and healthcare context. By sharing the model's successes and challenges, we identify practical lessons that may inform similar workforce‐development initiatives in other settings.

## 1 Introduction

Genomic technology is now part of routine healthcare worldwide, driving the expansion of the genetic counseling profession into regions like the Middle East and North Africa (MENA) (Abacan et al. 2019; Ormond et al. 2018, 2024). In recent years, local training programs have been established in the Kingdom of Saudi Arabia, Qatar, and the United Arab Emirates. Nevertheless, the MENA region continues to face a substantial gap between its high burden of inherited disease and the availability of culturally and linguistically competent genetic counseling services and local training programs (Shenbagam et al. 2025; Al‐Kharusi et al. 2024; Abiib et al. 2024). Many countries have historically relied on non‐local genetic counselors and interpreters, an approach that may not provide a sustainable basis for developing locally embedded services.

In Oman, a country of just over five million people (Omanis represent **~**57% of the population; expatriates **~**43%) (National Center for Statistics and Information 2026), the prevalence of genetic and congenital disorders exceeds 7%, over twice the global average (Modell and Darr 2002; Rajab et al. 2014; Al‐Kharusi et al. 2024). This high rate is largely due to prevalent consanguineous marriages, which have been reported to account for 52% of unions in Oman, increasing the risk of autosomal recessive disorders (Al‐Gazali and Hamamy 2014; Al Hoqani et al. 2021; Islam 2012; Rajab and Patton 2000; Tadmouri et al. 2009).

Despite this profound need, Oman's genetic counseling infrastructure developed only relatively recently. In 2011, Sultan Qaboos University Hospital (SQUH) established the first formal genetic counseling service. The lack of local academic training and limited workforce growth made it difficult to build educational infrastructure; as a result, a non‐Omani, non‐Arabic‐speaking, South African‐licensed genetic counselor initially led the service (Al‐Kharusi et al. 2024). This arrangement introduced immediate sociocultural and clinical challenges. Relying on interpreters often loses cultural nuance and reduces patient engagement, as interpreters may struggle with complex genomic concepts and psychosocial subtleties (Flores 2005; Ault et al. 2019; Bouska et al. 2025; Sheri 2022). Such barriers can hinder patient understanding and autonomy. In addition, a single trained genetic counselor could not meet Oman's national demand (Al‐Kharusi et al. 2024). Similar workforce and training constraints have been reported internationally, particularly in settings where the profession remains emergent (Abacan et al. 2019; Ormond et al. 2018).

## 2 Approach and Scope

This professional issue report provides a retrospective description and critical reflection on the Sultanate of Oman–University of Cape Town (UCT) in South Africa's training collaboration from 2015 to 2026. The authors reconstructed program characteristics from institutional records and the professional experience of those involved in program delivery, supervision, and participation. We drew workforce and service‐volume figures from previously published data and institutional records. We conducted no primary student interviews, validated outcome measures, formal exit surveys, or comparative statistical analyses. Accordingly, we present observations on student adjustment, progression, and program effectiveness descriptively and do not interpret them as causal findings.

## 3 The Transnational Split‐Funnel Model

### 3.1 Partnership Development and Division of Responsibilities

To address the absence of local genetic counseling training in Oman and support the profession's development, a transnational educational partnership was developed with the University of Cape Town (UCT) in South Africa (Al‐Kharusi et al. 2024). Traditional international education commonly requires students to relocate abroad full‐time, creating financial, logistical, linguistic, and sociocultural barriers, including visa restrictions, separation from support systems, and second‐language learning (Sabbadini et al. 2013; Nikam 2017; Akgumus et al. 2016; Vanneste et al. 2013). Students working in a second language may require additional time to develop the academic language proficiency needed for complex clinical communication (Choi 2005). Within genetic counseling training, second‐language use may further increase cognitive load because bilingual supervisees must develop discipline‐specific vocabulary while simultaneously acquiring advanced psychosocial counseling and communication skills (Vanneste et al. 2013).

The Transnational Split‐Funnel Model was developed to address these limitations by distributing complementary educational responsibilities between UCT and clinical institutions in Oman within a single UCT Master of Medical Science (MMedSc) program. The term “Split‐Funnel” reflects the allocation of training functions across two settings and their convergence toward a common outcome: preparing genetic counselors who met the academic and clinical requirements of an established master's program while developing the contextual competencies required for practice in Oman. UCT provided the curricular framework, concentrated academic and practical instruction, professional modeling, remote clinical facilitation, academic and clinical assessment, research supervision, and degree conferral. Omani institutions provided collaborative teaching and assessment support and longitudinal, supervised clinical training in Arabic and within the healthcare, cultural, and social contexts in which graduates were expected to practice. The model's distinguishing feature was therefore not international travel, block‐based teaching, or remote learning alone, but the integration of clinical training in Oman as a supervised and assessed component of the UCT degree. Regular clinical supervision, synchronous learning, collaborative academic activities, competency assessment, and paired research supervision maintained continuity between the two settings. This configuration enabled established academic infrastructure to support locally embedded clinical development and the gradual establishment of a national workforce. Table 1 compares the model with traditional international and conventional block‐release training, while Figure 1 illustrates the distribution and integration of training responsibilities.

**TABLE 1:** Comparison of traditional international training, conventional block‐release training, and the Transnational Split‐Funnel Model for genetic counseling education.

Dimension	Traditional international training	Conventional block release	Transnational Split‐Funnel Model
Academic teaching	Delivered mainly at an international institution after relocation.	Delivered in concentrated blocks separated by periods at the trainee's home site.	Concentrated UCT teaching and assessment, supplemented by synchronous learning while trainees were in Oman.
Clinical training	Conducted mainly in the host country and healthcare system.	Workplace experience occurs between academic blocks and may remain separate from institutional teaching.	Progressive training occurred at UCT and longitudinally in Oman as part of the same degree.
Supervision	Primarily provided at the host institution.	Usually provided locally between academic blocks.	Local supervision was integrated with continuing UCT oversight, joint learning, assessment, and paired research supervision.
Cultural and linguistic embedding	Adaptation to the home setting generally follows training.	Trainees remain connected to their workplace, but contextual adaptation may not be explicitly assessed.	Counseling competencies were developed in Arabic within Omani clinical and sociocultural contexts.
Relationship between sites	The international institution is the principal academic and clinical training site.	The academic institution provides teaching blocks, while the home site provides experience between blocks.	UCT and Omani sites held differentiated but interdependent roles within one longitudinal curriculum.
Goal	Education within an established international program.	Access to education while remaining connected to the home setting.	Preparation of locally practicing counselors who met UCT requirements while supporting professional capacity in Oman.

*Note:* These categories are operational definitions used for this comparison; individual international and block‐release programs may vary.

**FIGURE 1:**
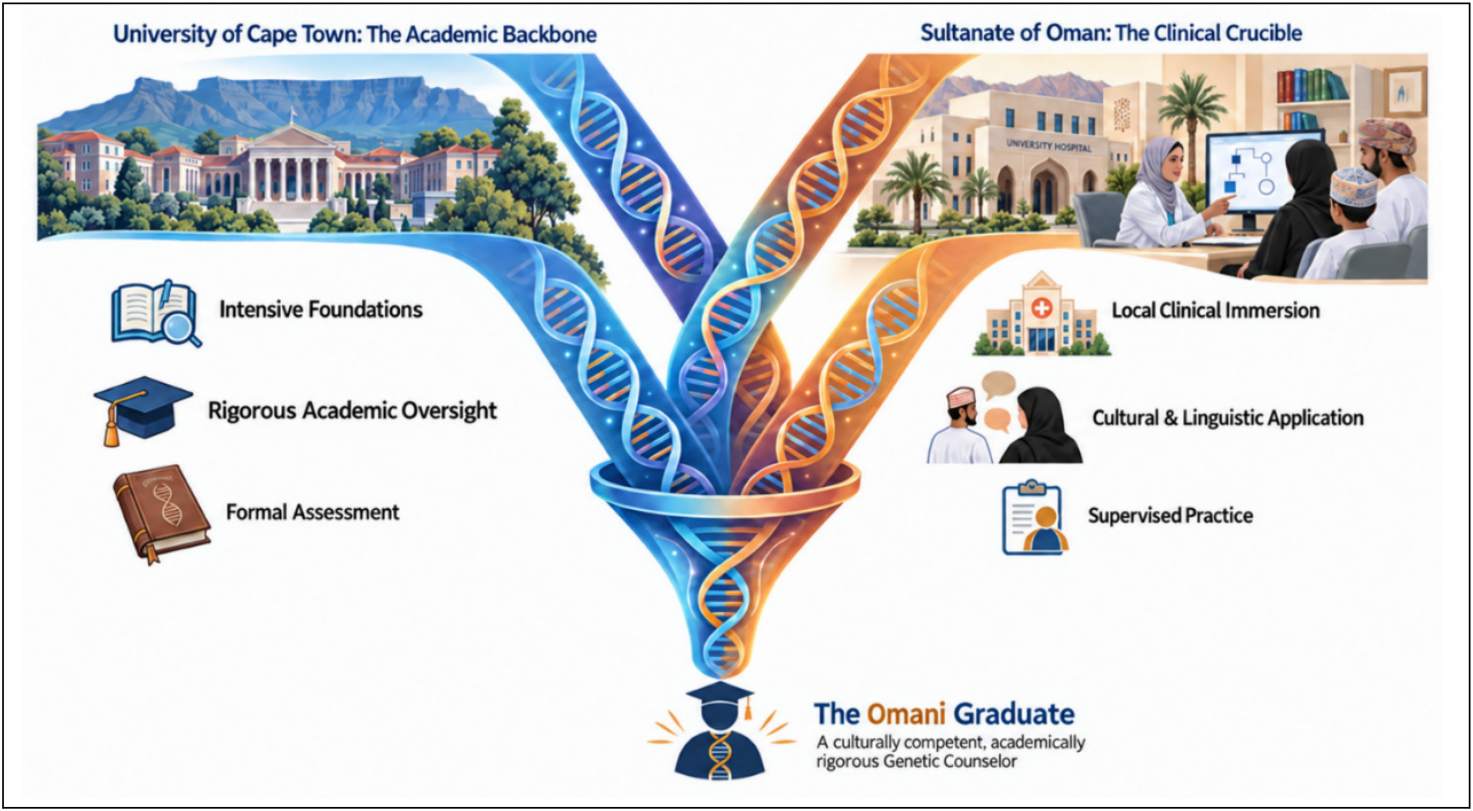
Schematic of the Transnational Split‐Funnel Model. UCT integrated UCT‐based teaching, assessment, and professional modeling with longitudinal clinical training in Oman through remote learning, cross‐institutional supervision, and dual research supervision.

The partnership emerged from bilateral negotiations between SQUH and UCT in 2013. The first Omani candidate completed the recommended prerequisite short‐course blocks in South Africa in March and August 2014 and enrolled in the MMedSc program in February 2015. Between 2015 and 2026, five Omani genetic counselors graduated through the model. Enrollment was initially limited to one international student per cohort and later increased to two, in alignment with available teaching and supervisory capacity.

### 3.2 Admission and Employment Criteria

To maintain academic rigor and professional alignment, UCT evaluated Omani candidates against its standard criteria for international postgraduate applicants. Eligibility required a relevant bachelor's degree, formal academic credential verification by the South African Qualifications Authority, and proof of English language proficiency with an overall International English Language Testing System score of 6.0. While UCT's selection process for South African applicants remains highly competitive, the Omani intake was intentionally limited to one to two students per cohort to align with available clinical supervision resources in South Africa and Oman.

UCT coordinated academic admission with, but kept it administratively separate from, employment recruitment in Oman. Before starting their genetic counseling studies, candidates had to secure positions as “genetic counselors‐in‐training” at Omani healthcare institutions. The local recruitment process included a comprehensive review of academic and professional credentials, a formal interview, and, in some clinical settings, an objective structured clinical examination and a probationary period. Thus, the local selection process complemented and was aligned with UCT's assessment of academic eligibility while independently addressing the workforce requirements of employing institutions. Employing institutions sponsored trainees throughout both the pre‐enrollment coursework and formal MMedSc program, covering tuition, travel, accommodation, and living expenses.

### 3.3 Preparatory Pathway

An additional component of the training pathway allowed candidates to complete selected degree courses before formally entering the master's program. The first candidate undertook the semester courses on the basic principles of genetic counseling and medical genetics in 2014. These didactic courses, which lacked a practical clinical element, were originally intended as mandatory prerequisites for Omani applicants. However, as local institutions prioritized rapid workforce development, this phase became a highly recommended but non‐compulsory preparatory step. Participants engaged in academic activities, including seminars and written coursework, alongside enrolled South African students. Following formal enrollment in the MMedSc program, completed coursework was recognized and credited toward the degree. This reduced the subsequent didactic workload and allowed trainees to spend more time on clinical rotations and research.

Importantly, this pre‐enrollment experience was not intended as a standardized admissions assessment; semester course performance was not standardized or consistently documented. Instead, its value lay in orienting candidates to UCT's academic environment, expectations, and the broader South African context. It also exposed candidates to both the parallels and differences between the South African and Omani professional settings, laying an early foundation for cross‐system adaptation. Of the five UCT graduates, two completed the pre‐enrollment pathway. Retrospective observations suggest that this experience facilitated their initial transition. However, given the small cohort size and differing subsequent workloads, no causal inferences about academic progression can be drawn.

### 3.4 Academic and Clinical Structure

The model's use of short‐term residencies in South Africa (3 months per year, over 2 years), rather than a continuous two‐year relocation, allowed trainees to alternate between the two training environments. Upon admission, Omani trainees participated fully alongside their South African peers in didactic courses, case seminars, and online workshops, thereby fostering a collaborative learning environment. The structured schedule integrated concentrated academic instruction and professional modeling at UCT with longitudinal clinical development in Oman (Table 2). Two primary UCT genetic counseling faculty members delivered academic teaching, supported by the clinical genetics division. Exposure to established genetic counselors at UCT supported professional identity formation and a foundation in psychosocial counseling, while practice at SQUH and SQCCCRC with the National Genetics Center (NGC) at Royal Hospital included in 2023 enabled trainees to develop counseling competencies in Arabic and within the Omani healthcare context.

**TABLE 2:** Academic and clinical structure of the Transnational Split‐Funnel Model.

Period	Location and delivery	Modules and activities	Clinical progression and supervision
Year 1: Academic and clinical initiation (up to 3 months per year)	UCT, Cape Town; in‐person teaching and assessment	*Medical Genetics 1 and 2*; *Principles of Genetic Counseling 1 and 2*; lectures, seminars, assignments, and examinations	Observerships at UCT‐affiliated hospitals, progressing to family‐history collection under direct and then indirect supervision
Year 1: Longitudinal clinical training	SQUH and SQCCCRC (NGC from 2023), Oman	*Practices of Genetic Counseling 1*	Translation, pedigree collection, information provision, and progression toward supervised counseling
Year 2: Academic and clinical rotation (up to 3 months per year)	UCT, Cape Town; in‐person teaching and assessment	Seminars, assignments, clinical rotations with written and clinical examinations	Continued observership and Supervised counseling
Year 2: Longitudinal clinical training	SQUH and SQCCCRC (NGC from 2023), Oman	*Practices of Genetic Counseling 2*	Supervised counseling of increasingly complex cases; supplementary rotations when required before clinical exit assessment
Across the program	Synchronous collaboration between Oman and South Africa	Weekly case supervision; journal clubs approximately every 2 weeks; monthly role‐play; departmental seminars approximately every 2 months	Local clinical supervision combined with remote UCT oversight, and continuing assessment
		Research development tutorials and supervision	paired UCT–Oman research supervision

*Note:* The frequency of synchronous activities varied by cohort and training stage.

Research supervision paired a UCT genetic counselor with an Omani clinical co‐supervisor to maintain academic standards while addressing locally relevant healthcare questions. Continuing cross‐institutional supervision and collaborative learning connected the two settings, distinguishing the model from block release in which academic and workplace components may operate separately.

## 4 Implementation Challenges and Program Adaptations

International students who relocate for their entire program face substantial financial and administrative burdens (Akgumus et al. 2016; Nikam 2017). The Transnational Split‐Funnel Model similarly required international travel, visa processing, and cross‐institutional coordination; however, shorter periods in South Africa were intended to reduce living costs and disruption to employment and family life.

Trainees furthermore faced substantial competing demands. Many worked full‐time as biomedical scientists while serving as genetic counselors‐in‐training under the direct supervision of genetic counselors and clinical geneticists (Al‐Kharusi et al. 2024). In addition to these concurrent employment, academic, and clinical responsibilities, trainees managed family and community obligations. Comparable social, psychological, and academic challenges, including adaptation to unfamiliar healthcare systems and educational expectations, have been reported among international students undertaking genetic counseling and other clinical training (Sabbadini et al. 2013; Attrill et al. 2016). In the transnational model, these pressures were compounded by the requirement to navigate two educational and healthcare systems simultaneously, meet international academic standards, and remain embedded in the local clinical and cultural environment. Notably, program coordinators reported no significant challenges related to differing learning styles between cohorts, although this was not formally evaluated.

Limited local supervisory capacity presented an additional challenge. The partnership was initially anchored by the South African‐trained expatriate genetic counselor who established Oman's first formal genetic counseling service at SQUH in 2011 and served as the sole on‐site clinical supervisor until 2021 (Al‐Kharusi et al. 2024), when she returned to South Africa. Her knowledge of South African academic standards and Omani clinical and cultural practices supported curriculum adaptation and early professional identity development. Between 2021 and 2026, supervisory capacity at SQCCCRC expanded further through the appointment of two professionals trained and licensed in South Africa: a genetic counselor and a clinical geneticist.

Across the 11‐year timeline, enrollment was staggered to align with supervisory resources, as summarized in Figure 2. Supervisory capacity expanded through the expansion of services at SQCCCRC, recruitment of an Omani Cardiff graduate at the NGC, and transition of the first Omani graduate into a faculty role. After 2022, supervision had expanded from one expatriate counselor to a multi‐site network of three genetic counselors supported by nine clinical and biochemical geneticists with international subspecialty training. After 5 years of independent clinical practice, the first Omani graduate transitioned into a faculty role. The graduate undertook supervision as part of the wider faculty and multi‐site supervisory network rather than as an independent sole supervisor. Supervisory readiness was determined through institutional appointment and credentialing processes, supported by faculty‐development activities in clinical supervision, feedback, and workplace‐based assessment. The model maintained a 1:1 trainee‐to‐supervisor ratio throughout.

**FIGURE 2:**
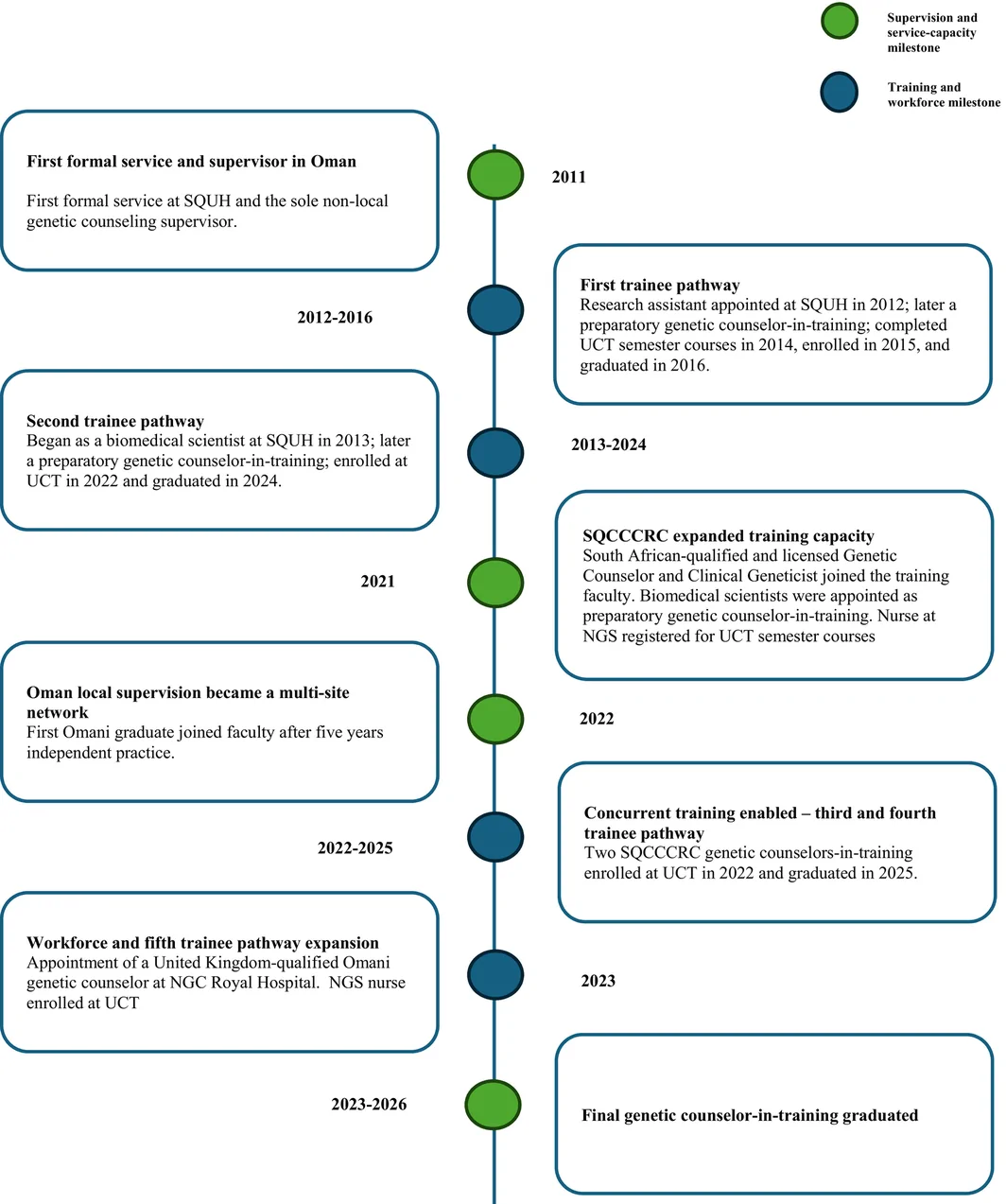
Development of genetic counseling training and supervision capacity in Oman, 2011–2026. The timeline shows the staggered trainee pathways, expansion of clinical sites and workforce, and progressive development of local supervisory capacity.

Institutional credentialing and faculty‐development processes supported teaching and supervision at both sites. For genetic counselor supervisors, teaching and supervision formed part of their salaried institutional responsibilities; no additional compensation was provided specifically for these activities. UCT faculty and supervisors were subject to university appointment requirements, Health Professions Council of South Africa licensure standards, and continuing professional development requirements (HPCSA 2013). At SQUH, clinical staff were formally required to participate in teaching, learning, and student assessment. At SQCCCRC, a senior genetic counselor with qualifications and experience in health professions education supported clinical training. Omani faculty and supervisors also participated in development programs offered by the Oman Medical Specialty Board and local genetics fellowship programs, focusing on clinical supervision, feedback, and workplace‐based assessment.

Rather than reflecting academic failure or attrition, several trainees required program extensions because of personal circumstances, research delays, or the need for additional clinical exposure. Three trainees required six‐month research extensions associated with pregnancy and childbirth. COVID‐19 restrictions also delayed data collection and research progression for at least one trainee. Research completion was further affected by the time required to obtain ethics approvals in both Oman and South Africa, the demands of qualitative data collection and analysis, and the need to balance study with employment and family responsibilities. These extensions represented individualized adjustments to completion timelines rather than academic remediation.

Supplementary clinical training was required when trainees had not yet obtained sufficient breadth of clinical exposure. Two trainees, based primarily at the specialist oncology center, developed substantial experience in cancer genetics but had fewer opportunities to gain the required exposure to general pediatric and prenatal genetics. They therefore registered for an additional academic year and completed a six‐month supervised general‐genetics rotation before undertaking their clinical exit examinations. The additional rotation, supervised by senior genetic counselors and clinical geneticists, was intended to ensure that their overall clinical experience met the breadth expected of program graduates.

Although the department did not conduct formal trainee interviews or quantitative exit surveys, the departmental board and program director periodically discussed trainee issues and implementation challenges. This informal monitoring identified recurring logistical and workload‐related concerns. The intensive UCT academic and clinical blocks were demanding for trainees and supervisors. Other challenges included complex international registration processes, limited employer‐sponsored funding, work‐release arrangements, and prolonged visa delays. In response, the program strengthened communication regarding dual enrollment, expanded local supervisory capacity to reduce pressure on South African placements, and engaged earlier with the UCT international student office. Table 3 summarizes the principal implementation challenges, program adaptations, residual limitations, and lessons for future programs.

**TABLE 3:** Implementation challenges and program adaptations.

Implementation challenge	Program adaptation implementation	Residual limitation	Lesson for future programs
Distributed academic and clinical sites	Cross‐site supervision and scheduled online activities	Frequency varied across cohorts	Establish a formal schedule, minimum frequency, and clearly defined responsibilities for cross‐site supervision and learning activities.
Competing employment and family responsibilities	Individualized timelines and extensions	No formal evaluation of effectiveness	Incorporate protected training time and flexible progression pathways, with prospective evaluation of their effects on trainee progression and outcomes.
Limited general‐genetics exposure in a specialist center	Six‐month supplementary rotation	Extended time to exit assessment	Map the local case mix against required competencies before training begins and arrange supplementary placements early.
Research delays	Continued supervision and revised milestones	Ethics and data‐collection delays remained	Develop feasible research timelines and initiate ethics, institutional, and data‐access processes as early as possible.
International logistics	Employer sponsorship and early administrative liaison	Visa and work‐release barriers persisted	Confirm funding, visas, travel arrangements, and protected work release before enrollment, with contingency plans for delays.

Because the analysis was reconstructed primarily from institutional records and reflections of individuals involved in program development and supervision, it privileges institutional and educator perspectives. Informal monitoring cannot substitute for systematically elicited trainee accounts. Future evaluation should directly examine the perspectives of graduates, supervisors, patients, and institutional stakeholders.

## 5 “System Code‐Switching” in Clinical Training

We use “system code‐switching” to describe how trainees translated and adapted professional knowledge and counseling practices across different linguistic, cultural, ethical, educational, and healthcare systems while retaining the core principles of genetic counseling.

Trainees absorbed theoretical frameworks and counseling models in English within the South African academic context, then translated and adapted them to Arabic within Omani social, cultural, and religious frameworks. This friction is consistent with findings by Altemura et al. (2024), who note that translating Western, emotion‐centered counseling constructs into other languages can lead to cultural discomfort and unmet clinical expectations. As a result, trainees had to continuously adapt, not just linguistically, but also in ethical reasoning, communication styles, and models of care, mirroring broader principles of cultural competence and cross‐cultural clinical adaptation (Betancourt et al. 2002; Kleinman and Benson 2006).

In conventional long‐term international models, students are often fully assimilated into the host country's healthcare system and experience significant “re‐entry shock” upon returning home. Our model, by contrast, employed a brief, intensive three‐month academic placement in South Africa, interspersed with extended clinical immersion in Oman. This cyclical structure created repeated opportunities to compare, apply, and discuss learning across the two settings. During their academic and clinical blocks in South Africa, trainees were immersed in a well‐established, highly structured genetic counseling environment. They observed senior clinicians, conducted counseling sessions, participated in in‐person assessments, and anchored their professional identity within a mature peer community, establishing clear clinical benchmarks. Upon returning to Oman, trainees immediately entered “the clinical crucible,” actively translating and adapting South African benchmarks to the collectivist, Arabic‐speaking Omani setting.

To help students navigate these transitions, the program implemented several structured scaffolding mechanisms. Academic and clinical mentors at both sites maintained continuous, synchronous online communication, including weekly clinical case supervision. When trainees in Oman faced clinical or cultural dilemmas, such as restricted reproductive options or counseling large, consanguineous families, they could immediately consult with both UCT and local mentors for reflective guidance. Omani clinical sites also provided on‐the‐ground support through local geneticists and senior counselors who modeled clinical code‐switching. In addition, role‐playing workshops in South Africa anticipated these transitions, allowing students to practice counseling scenarios in English and Arabic before returning to Omani clinics. This cross‐border mentorship provided structured support as trainees adapted their learning between the two settings.

The practical significance of “system code‐switching” was most apparent in three domains. First, trainees learned counseling approaches in English but delivered most clinical care in Arabic. This required more than literal translation: they had to communicate genetic risk, uncertainty, stigma, and emotional concerns in culturally and linguistically appropriate ways. Although trainees initially anticipated reluctance toward psychosocial discussion, their programmatic experience suggested that patients disclosed grief, fear, and uncertainty when counselors created appropriate opportunities for emotional exploration. This observation was not formally evaluated but indicated that counseling techniques required adaptation rather than omission.

Second, reproductive counseling differed substantially between the two settings. Termination of pregnancy for serious congenital abnormalities is legally available in South Africa (Malope et al. 2023), whereas reproductive decision‐making in Oman occurs within more restrictive legal parameters and an Islamic bioethical framework (Al Aqeel 2007; Bruwer et al. 2022; Mustafa 2013). Trainees therefore had to adapt discussions of reproductive options to locally available and ethically permissible pathways while maintaining accurate information provision, informed choice, and psychosocial support.

Third, trainees navigated different expectations concerning participation in decision‐making. Although South African training foregrounded individual autonomy, decision‐making was not necessarily isolated; patients often considered the views of partners, extended family members, or their communities while retaining final decision‐making authority (Malope et al. 2023). Omani consultations more frequently involved couples and extended family members, reflecting the influence of collectivist values, extended family structures, and religious considerations (Al Marri et al. 2025). Trainees therefore had to manage consultations involving multiple participants without assuming that either individual or family‐centered decision‐making represented every patient's preference. Consistent with calls for locally focused and patient‐oriented genetic counseling (Weil et al. 2024), trainees learned to establish each patient's preferences regarding family involvement while protecting confidentiality and voluntary participation. Across these domains, “system code‐switching” involved preserving the core principles of genetic counseling while adapting their communication and application to the Omani linguistic, ethical, legal, and family context.

## 6 Model Outcomes, Broader Implications, and Future Directions

### 6.1 Academic Progression

We used available program records to describe enrollment, completion, and progression over the 11‐year period from 2015 to 2026. During this time, five Omani candidates enrolled in the UCT MMedSc program and followed the Transnational Split‐Funnel Model; all graduated, resulting in a 0% attrition rate.

### 6.2 Workforce and Service Outcomes

During the period in which the distributed transnational training model operated, Oman experienced measurable growth in the capacity of the genetic counseling workforce and in service delivery. As quantified by Al‐Kharusi et al. (2024), genetic counseling services expanded from a single non‐local‐led clinic in 2011 to three major national centers staffed by highly trained, culturally and linguistically competent Omani professionals. This workforce expansion was accompanied by rapid growth in service utilization (Al‐Kharusi et al. 2024).

After completing the degree, graduates entered a period of supervised practice in Oman. This locally delivered transition‐to‐practice period drew on Health Professions Council of South Africa internship expectations and Omani clinical training structures but was distinct from the academic requirements of the MMedSc degree. Graduates remained salaried employees of their Omani healthcare institutions and initially managed cases under direct supervision, progressing gradually toward more independent practice. Although salaried positions and service expansion demonstrate the operational integration of genetic counselors within participating institutions, the program did not formally evaluate how genetic counselors were perceived or accepted by other healthcare professionals, patients, or institutional stakeholders. These developments should therefore not be interpreted as evidence of profession‐wide acceptance.

### 6.3 Lessons for Learner Preparation and Support

Experience with this transnational model shaped institutional perspectives on student preparation, support, and programmatic design. While academic achievement, scientific knowledge, communication skills, and empathy remained essential, trainees also had to manage concurrent employment, family commitments, international travel, and frequent transitions across diverse academic and healthcare environments. We observed that trainees consistently demonstrated adaptability, resilience, initiative, persistence, and flexibility. Because these attributes were not formally assessed, we cannot conclude that they predicted success. Nevertheless, our retrospective experience suggests that these attributes helped trainees navigate the program and highlights the potential value of deliberately fostering them through preparation, mentoring, and flexible support throughout genetic counseling training. These demands are consistent with the broader health professions education literature, which indicates that international and culturally and linguistically diverse trainees may experience acculturative stress and difficulties navigating unfamiliar academic expectations, clinical environments, communication demands, and supervisory relationships (Attrill et al. 2016; Jeong et al. 2011; Junious et al. 2010; Mori 2000). Within genetic counseling education, international trainees have similarly described adjustment to unfamiliar assessment practices, supervisory approaches, and psychosocial counseling expectations (Sabbadini et al. 2013; Akgumus et al. 2016).

### 6.4 Broader Implications and Transferability

Beyond its local implementation, this experience offers a potential conceptual contribution to genetic counseling workforce development. It illustrates the feasibility and potential value of a distributed transnational approach in a setting without local academic infrastructure, combining an established international academic program with context‐specific clinical training. Importantly, this model challenges traditional, location‐bound approaches to professional education by introducing a more flexible, distributed training paradigm. “System code‐switching” may represent a critical competency for the future genetic counseling workforce, particularly as care becomes increasingly globalized and culturally complex.

However, as this descriptive professional issues report reflects the experience of five trainees within one partnership, it does not establish the model's effectiveness, scalability, or transferability to other settings. Its emphasis on flexibility and regional adaptation is nevertheless consistent with McEwen et al. (2025), who highlighted the need for emerging genetic counselor education frameworks to be tailored to local healthcare priorities and cultural contexts.

### 6.5 Local Training and Regulation

For Oman, the next phase of development is establishing an independent master's‐level genetic counseling program at SQUH. Building on the framework submitted in 2023 (Al‐Kharusi et al. 2024), the program has received institutional approval, with implementation planning underway at the time of writing. It is intended to operate as an independently accredited Omani program rather than as a joint degree with a South African university. Continued collaboration with UCT is being considered for activities such as external curriculum review, research co‐supervision, and external examination; however, the form and scope of this collaboration have not yet been formalized.

This planned transition is consistent with Paneque et al.'s (2026) description of professional development as progression through early, emerging, established, and consolidated stages. Within this framework, the development of local education, regulation, and professional capacity represents an important transition from reliance on international support toward a more established and sustainable national profession.

A key challenge for the next phase of workforce development is formal regulatory recognition of genetic counseling as a distinct profession, a difficulty reported internationally in countries where the profession remains emergent (Ormond et al. 2018, 2024). Regional developments in Qatar and the United Arab Emirates, including the introduction of professional requirements and defined scopes of practice, provide relevant examples for Oman as it develops its own regulatory pathway (Abiib et al. 2024; Department of Health Abu Dhabi, 2024). Continued professional development will also depend on access to locally relevant genomic evidence. Regional genome initiatives may strengthen the evidence base for providing clinically appropriate genetic counseling for Middle Eastern populations.

## 7 Conclusion

The Oman–UCT collaboration illustrates how established international academic infrastructure can support the development of a locally embedded genetic counseling workforce while domestic training capacity is being established. The Split‐Funnel Model purposefully distributed and longitudinally integrated academic teaching, clinical training, supervision, assessment, research, and professional development across UCT and clinical training sites in Oman. Through this collaboration, five Omani trainees completed master's‐level education while developing counseling competencies within the linguistic, cultural, and healthcare contexts in which they were expected to practice.

The experience also demonstrates the practical complexity of distributed transnational training. International logistics, concurrent employment and family responsibilities, research delays, variable clinical exposure, and limited supervisory capacity required staggered enrollment, cross‐site supervision, flexible completion timelines, supplementary clinical placements, and sustained employer support. Within this structure, system code‐switching captured how trainees preserved the core principles of genetic counseling while adapting their application across the South African and Omani settings.

Because this report reflects the retrospective experience of the transnational training partnership, further evaluation is needed before conclusions can be drawn about the model's effectiveness, scalability, or transferability. Its contribution is therefore not a universally applicable training template, but a documented example of how academic resources in one country may be combined with supervised, context‐specific clinical development in another. For Oman, the model represents a step in the transition from reliance on international academic support toward independent local education, professional regulation, and sustainable workforce development.

## Author Contributions

**Chantel van Wyk:** conceptualization, writing – original draft, visualization, writing – review and editing, resources, data curation, investigation, validation, formal analysis, project administration. **Zandre Bruwer:** conceptualization, investigation, writing – review and editing, validation, formal analysis. **Khalsa Al‐Kharusi:** conceptualization, investigation, validation, writing – review and editing, formal analysis. **Nakita Laing:** validation, visualization, formal analysis, project administration. **Tina‐Marié Wessels:** conceptualization, investigation, validation, visualization, writing – review and editing, formal analysis, project administration, resources.

## Ethics Statement

This manuscript is a reflective piece on a professional training program and does not report on human subjects' research; therefore, specific ethics committee approval was not required.

## Consent

This manuscript is a reflective piece on a professional training program and does not report on human subjects' research; therefore, specific patient informed consent was not required.

## Conflicts of Interest

The authors declare no conflicts of interest.

## Data Availability

Data sharing is not applicable to this article as no new primary quantitative or qualitative data were generated or analyzed for this reflective professional issues report. Additional program information may be available from the corresponding author on reasonable request.
